# Optimized total thoracoscopic and laparoscopic esophagectomy for esophageal cancer

**DOI:** 10.1186/s12957-016-0824-6

**Published:** 2016-03-09

**Authors:** Shao-hui Zhou, Yong-bin Song, Li-jun Liu, Hong-shang Cui

**Affiliations:** Department of Thoracic Surgery, Hebei Provincial People’s Hospital, Shijiazhuang, 050051 China

**Keywords:** Esophageal cancer, Esophagectomy, Laparoscopy, Thoracoscopy, Complication

## Abstract

**Background:**

Total thoracoscopic and laparoscopic esophagectomy (TLE) has attracted attention with the advantage of better operative field and minimal wound for the esophageal cancer. However, various severe complications are also reported during the TLE such as cervical anastomotic leakage, chylothorax, and tracheal injury. The aim of this study was to introduce a new optimized TLE procedure for the esophageal cancer and assess its safety and clinical effects.

**Methods:**

We retrospectively collected the clinical data of 30 patients with esophageal cancer who underwent optimized TLE procedures between January 2014 and December 2014. The optimized TLE procedures mainly include as follows: (1) 50 ml of sesame oil-milk mixture (1:1) is injected via gastric tube after endotracheal intubation; (2) patients are intubated with a single lumen endotracheal tube; (3) patients were positioned at 150° in the left prone position rather than lateral decubitus position; and (4) duodenal feeding tube was not placed intraoperatively and however triple lumen nasojejunal feeding tube was placed on the second postoperative day under imaging guidance. Operation time, amount of blood loss, number of dissected nodes, length of hospital stays, and complications were recorded.

**Results:**

The mean operation time of the optimized TLE group was 202.13 ± 13.74 min. The mean visible blood loss of the optimized TLE group was 300.00 ± 120.12 ml. The postoperative hospital stays in the optimized TLE group were 16.27 ± 4.51 days. The number of dissected nodes in the optimized TLE group was 13.57 ± 2.76. The postoperative complications for the optimized TLE procedure were seen in one case (3.3 %).

**Conclusions:**

The method of optimized TLE is an effective, reliable, and safe procedure for the treatment of esophageal cancer, which provide favorable outcomes in terms of operation time, blood loss, length of hospital stays, the number the dissected nodes, and reduced incidence of postoperative complications compared to previous literatures. Further studies with a large number of samples are warranted.

## Background

China has a high incidence of esophageal cancer, with a mortality rate ranking fourth of all malignant tumors [1]. Esophageal resection is one of the primary therapeutic methods for the esophageal cancer [2]. However, because of the relatively high mortality and morbidity associated with esophagectomy for esophageal cancer [3–5], there have been many efforts to reduce its invasiveness [3, 6, 7]. Minimally invasive esophagectomy (MIE) has become increasingly used and been accepted with reported outcomes comparable with those of open approaches [8–13]. The techniques used can be thoracoscopic, laparoscopic, or both. As the experience and skills for thoracoscopic and laparoscopic surgery improve, total thoracoscopic and laparoscopic esophagectomy (TLE) has attracted more and more attention with the advantage of better operative field and minimal wound [14–18]. However, various severe complications are also reported during the TLE such as cervical anastomotic leakage, chylothorax, and tracheal injury. Thus, how to avoid surgical risks and improve surgical efficiency further has become the focus. The aim of this study was to introduce an optimized TLE procedure for esophageal carcinoma and assess its safety and outcomes.

## Methods

### Patients

The study was approved by the Ethics Committee of the Hebei Provincial People’s Hospital and conducted in accordance with the Declaration of Helsinki. Written informed consent was obtained from all subjects. We retrospectively evaluated 30 patients with esophageal cancer who underwent optimized thoracoscopic and laparoscopic esophagectomy between January 2014 and December 2014. Histological diagnosis of esophageal cancers was confirmed by esophagoscopy in all patients prior to surgery. The inclusion criteria were (1) patients with clinical stage I, II, and IIIa and IIIb esophageal cancer. The exclusion criteria were (1) patients with clinical stage IIIc and IV esophageal cancer; (2) patients with dyscrasia, or severe heart, liver, brain, and kidney concomitants, (3) patient who cannot subject one-lung ventilation due to poor lung function, and (4) patients have history of thoracic and abdominal surgery. The clinical characteristics of the 30 cases were summarized in Table 1.

**Table 1:** Baseline characteristics

	Optimized TLE procedure (*n* = 30)
Age (years), mean ± SD	65.63 ± 3.71
Sex	
Male	21 (70 %)
Female	9 (30 %)
Comorbidity	
Hypertension	3 (10 %)
Diabetes mellitus	2 (6.7 %)
COPD	1 (3.3 %)
Lacunar infarction	2 (6.7 %)
ASA score	
1	2 (6.7 %)
2	28 (93.3 %)
pStage	
I	2 (6.7 %)
IIa	4 (13.3 %)
IIb	6 (20.0 %)
IIIa	11 (36.7 %)
IIIb	7 (23.3 %)
Tumor location	
Upper	16 (53.4 %)
Middle	13 (43.3 %)
Lower	1 (3.3 %)
Histology	
Squamous carcinoma	28 (93.3 %)
Adenocarcinoma	2 (6.7 %)

*ASA* American Society of Anesthesiologists, *COPD* chronic obstructive pulmonary disease

### Preoperative evaluation

Preoperative work-up included blood routine examination, urine routine examination, blood biochemical test, blood coagulation test, electrocardiogram, echocardiography, chest and abdominal computed tomography (CT) scans, endoscopic ultrasonography, positron emission tomography-computed tomography (PET), upper gastrointestinal barium swallow, and pulmonary function test.

### Surgical technique

The surgeries were performed by a group of three surgeons who have more than 15 years’ surgical experience. Intrathoracic pressure of 8 mmHg and intraabdominal pressures of 12 mmHg were used for thoracic and abdominal surgery, respectively. The difference of conventional procedure and our optimized procedures was as follows:

During the conventional TLE procedure, the patient is often intubated with a double lumen endotracheal tube for single lung ventilation in the normal side and positioned in the left lateral decubitus position. The stomach contents are aspirated to the greatest extent to empty the stomach using a normal stomach tube before surgery. Whereas during our optimized TLE group, the patient is intubated with a single lumen endotracheal tube for bilateral lung ventilation and positioned in the prone position with a 150° left lateral tilt. After endotracheal intubation, 50 ml of sesame oil-milk mixture (1:1) is injected via gastric tube.

The procedure was conducted in three stages. Firstly, thoracoscopic dissection was done with the patient in the left lateral decubitus position in conventional group and prone position in the optimized group. Four thoracoscopic ports are placed. The l2-mm camera port is placed at the seventh intercostal space on the midaxillary line. Two 5-mm ports (shaft handle’s hole) are placed, one at the fifth intercostal space posterior to the posterior axillary line and one at the fourth intercostal space at the anterior axillary line. One additional 12-mm port (assistant handle hole) is placed at the eighth intercostal space, posterior to the posterior axillary line. The lung was retracted laterally using an atraumatic instrument. Ultrasound knife or electrocautery was used to separate the mediastinal pleura overlying the esophagus to detect whether there exists the extra-esophagus invasion such as thoracic aorta, membranous part of the trachea, and left main bronchus. The azygos vein was divided using Hemolok clips (Weck; Teleflex Medical, Durham, NC, USA), and then the esophagus was circumferentially mobilized from the esophageal hiatus up to the thoracic inlet. Paraesophageal lymph nodes were dissected and removed or mobilized en bloc with the resected specimen. Then complete hemostasis was performed using hook electrode, and the chest cavity was flushed with normal saline. A 32F chest tube was inserted through the camera port for postoperative chest drainage.

In the second stage, the patient was placed in the supine position. The surgeon stands on the patient’s right side and the assistant on the left side. Five abdominal ports were placed on the anterior abdominal wall. The l2-mm camera port is placed at 1 cm superior to umbilicus. Two shaft handle’s ports are placed (12- and 5-mm), one at 5 cm lateral to the lateral border of the rectus abdominis muscle and one at the junction between the right midclavicular line and costal arch. Two additional 5-mm ports (assistant handle hole) are placed, one at the epigastrium, approximately 3 cm below the xiphoid process and one at the junction areas between the costal arch and the left anterior axillary line. The operator stands on the right side of the patients. After detecting the abdominal cavity, the entire greater curvature of the stomach was mobilized, preserving the right gastroepiploic vessels. The left gastroepiploic artery, short gastric artery, and the peritoneum overlying the abdominal esophagus were isolated and divided with an ultrasound knife (HARMONIC ACE36E, Johnson & Johnson, New Brunswick, NJ, USA). With a bowel forceps, the left lobe of the liver is retracted upward to expose the gastrohepatic ligaments and abdominal esophagus. The lesser omentum was dissected with ultrasound knife. The gastrohepatic ligament, the peritoneum overlying the abdominal esophagus and the diaphragm’s esophageal hiatus, was divided. Left gastric vessels overlying the pancreas superiorly were retracted with the endoscopic bowel forceps, and then the two ends of left gastric vessels were clamped and divided with ligature forceps. Lymph nodes around the left gastric artery, splenic artery, and common hepatic artery were resected. After the bilateral crus of the diaphragm is exposed, the abdominal esophagus is retracted to the abdominal cavity. The pneumoperitoneum was removed, and incision below the xiphoid cardiac was expanded approximately 3 cm. The gastric tube was then created by dividing the stomach starting at the distal lesser curve and ending at the cardiac notch (angle of His) using the linear cutting staplers, preserving the right gastric vessels. The gastric tube was then sutured to prevent twisting and the mobilized esophagus for tunneling to the neck using the interrupted seromuscular sutures.

In the third stage, a 4-cm horizontal neck incision is made just above the suprasternal notch, and then the cervical esophagus is exposed. The cervical esophagus is divided, and the esophagogastric specimen was pulled out of the abdomen incision. As traction is applied to the specimen in the neck, another surgeon guides the specimen in its proper alignment into the mediastinum. The specimen is removed from the field. An anastomosis is performed between the esophagus and gastric tube with PROXIMATE ILS Curved and Straight Intraluminal Staplers (Ethicon Endo-Surgery, LLC). The duodenal feeding tube and stomach tube were placed across the anastomosis. Fundus of the gastric tube was excised using a linear cut stapler, and the margin was closed with interrupted seromuscular suture in a figure-of-8 pattern using non-absorbable Sutures Mersilk (braided silk).

### Postoperative care

The differences of our optimized procedures and conventional optimized procedures were as follows:

During conventional procedures, patients started nasogastric nutritional support with duodenal feeding tube and gastrointestinal decompression with stomach tube on the second postoperative day due to gastric emptying time which is about 24–48 h after gastroenteric surgery. Oral diet was started after duodenal feeding tube, and stomach tube was removed on the fifth postoperative day.

During optimized group, patients started nasogastric nutritional support with triple lumen nasojejunal feeding tube (Freka-Trelumina; Fresenius, Bad Homberg, Germany) on the second postoperative day. Distal intestinal feeding tube was passed from pylorus to the jejunum through Treitz’s ligament. Oral diet was started after triple lumen nasojejunal feeding tube was removed on the 10th postoperative day.

### Statistical analysis

Data analyses were performed using SPSS version 17.0 for windows (SPSS Inc., Chicago, IL, USA). All data are expressed as the mean and standard deviation and number or percentage as appropriate.

## Results

The patients consisted of 21 males and 9 females. The mean age was 66 years (range, 56–72 years). The characteristics of 30 patients undergoing optimized TLE for esophageal cancer with regard to age, sex distributions, incidence of comorbidities, tumor location, histology except preoperative staging, and American Society of Anesthesiologists (ASA) grade are shown in Table 1.

Surgical outcomes are shown in Table 2. The mean total operation time for the optimized procedures was 202.13 ± 13.74 min. The mean blood loss was 300.00 ± 120.12 min. The postoperative hospital stays for the optimized procedures were 16.27 ± 4.51 days. The number of total lymph nodes removed was 13.57 ± 2.76 per patient in the optimized TLE group. Our perioperative outcomes were slightly better compared with the previous literatures [17–21].

**Table 2:** Surgical outcomes of patients receiving optimized TLE procedures

	Optimized procedure (*n* = 30)
Operation time (min)	202.13 ± 13.74
Blood loss (ml)	300.00 ± 120.12
Length of hospital stay (days)	16.27 ± 4.51
Number of dissected nodes	13.57 ± 2.76

With regard to the postoperative complications, the cervical anastomotic leakage was seen in one case (3.3 %) in our series. No other complications such as chylothorax, tracheal fistula, recurrent laryngeal nerve injury, and atelectasis were observed (Table 3). Our findings were significantly lower than the previous literatures [1, 14, 17, 18, 20–22].

**Table 3:** Postoperative complications of patients receiving optimized TLE procedures

	Optimized procedure (*n* = 30)
Postoperative complications	1 (3.3 %)
Cervical anastomotic leakage	1 (3.3 %)
Chylothorax	0
Tracheal fistula	0
Recurrent laryngeal nerve injury	0
Atelectasis	0

## Discussion

Thoracoscopic esophagectomy is currently accepted by more and more thoracic surgeon due to minimal trauma and rapid recovery [19]. With the recent advance in thoracoscopic surgery, endoscopic esophagectomy gradually modified from the endoscopic-assisted mini-incision to total thoracoscopic and laparoscopic esophagectomy. This modification helps to the decrease of the surgical time and postoperative trauma [23]. However, with the number of the operation increase, some complications which rarely reported in the conventional three-port surgery also occur because the operations are more complex than those required for other malignancies [18, 24–27]. After reviewing the aforementioned literatures, we found the overall postoperative complications following TLE can reach up to about 47.6 % [17, 20, 28, 29]. Thus, how to reduce complications has become one issue that needs to be addressed urgently. We analyzed the clinical data of cases undergoing conventional TLE procedures in our hospital and found that most of the patients develop tracheal fistula 4–14 days after surgery when the gastric tube was removed and diet was begun. We consider that it may be due to the strong pressure difference between the negative intrathoracic and positive intraabdominal pressure. In addition, bilateral vagal neurectomy is liable to cause pyloric obstruction, which may enhance the pressure difference. Optimized TLE is a modification of conventional TLE, intended to diminish surgical trauma and decrease technical complications related to esophagectomy and associated lymph node dissection and reconstruction of the gastrointestinal tract, such as anastomotic leakage, recurrent laryngeal nerve palsy (RLNP), and chylothorax. In the current study, we optimized the protocols of the endoscopic esophagectomy. Patients receiving optimized TLE started nasogastric nutritional support with triple lumen nasojejunal feeding tube on the second postoperative day. Tubular stomach was maintained in a condition of negative pressure via cutting multiple-side holes into the gastric tube lateral wall. Distal intestinal feeding tube was passed from pylorus to the jejunum through Treitz’s ligament. Oral diet was started after triple lumen nasojejunal feeding tube was removed on the 10th postoperative day. These procedures resolved the difficulties such as functional obstruction at the pylorus, nutritional solution aggregation at the duodenum, and early postoperative enteral nutrition, and meanwhile reduce the surgical time. Our findings showed that the rate of postoperative complications in patients receiving the optimized TLE procedures was 3.3 %, which significantly improved compared to the previous reports.

In this study, patients in the optimized TLE group are intubated with a single lumen endotracheal tube for bilateral lung ventilation and positioned in the lateral prone position. Through these procedures, the operative field of the esophagus bed was similar in the conventional and optimized TLE group under the application of artificial pneumothorax. Due to the absence of two-lumen endotracheal tubes cuffs, the membranous part of the trachea is retrieved, which can clearly visualize lymph around the recurrent laryngeal nerve in the left side. In this study, patients treated with optimized TLE did not develop any recurrent laryngeal nerve injury. With regard to the number of retrieved total lymph nodes collected, our study demonstrated slightly higher number of lymph nodes dissected in the optimized TLE group compared to the previous reports.

Thoracic duct injury is a rare but serious complication following chest surgeries and major neck dissections, which may lead to nutritional deficiencies, respiratory dysfunction, and immunosuppression with a mortality up to 50 % in untreated patients [30, 31]. Clinically, it can present as cervical chylous fistula, chylothorax, or chylopericardium. As reported, 60~70 % of fat intake come together into cisterna chili via lymph vessels. Enterogenous lymph has a clinical presentation of milky white appearance due to a high content of triglycerides and chylomicrons. The flow and the characteristics of chyle from thoracic duct cannulae vary with the diet and usually about 60–100 ml/h. This flow will increase obviously with a presentation of chyle appearance after feeding (fat intake) and reduce with a presentation of clear appearance under hungry or fasting conditions. Patients should be fasted before the esophagectomy which go against the intraoperative distinguishment of the thoracic duct injury due to the clear lymphatic fluid in the thoracic duct. In the optimized TLE group, we infused the triglyceride and protein-rich oil-milk mixtures via gastric tube after tracheal intubation. Once the intraoperative thoracic duct injury occurs, it will accompany white fluid flow, which contributes to the prevention of the thoracic duct injury. In this study, we found no thoracic duct injury based on the above modifications of the TLE.

## Conclusions

Optimized TLE is a safety and efficient surgical modality for esophageal cancer, which provide favorable outcomes in terms of operation time, blood loss, length of hospital stays, the number the dissected nodes, and reduced incidence of postoperative complications compared to previous literatures. Further studies with randomized controlled trials are necessary for confirming the surgical efficacy of our technique.

## Abbreviations

ASA: American Society of Anesthesiologists
CT: computed tomography
MIE: minimally invasive esophagectomy
PET: endoscopic ultrasonography, positron emission tomography-computed tomography
RLNP: recurrent laryngeal nerve palsy
TLE: total thoracoscopic and laparoscopic esophagectomy
